# Exploring the impact of intensive versus standard blood pressure management following post-endovascular therapy in ischemic stroke: A comparative systematic review and meta-analysis

**DOI:** 10.5339/qmj.2025.21

**Published:** 2025-03-17

**Authors:** Vikash Kumar Karmani, Yusra Mashkoor, Anshahrah Riaz, Zunera Khalid, Bijay Mukesh Jeswani, Inshal Jawed, Hina Khan, Mohitha Chowdary Mallipeddi, Manisha Chavan, Ajay Singh, Shahzad Zafar

**Affiliations:** ^1^Department of Internal Medicine, Jinnah Sindh Medical University, Karachi, Sindh, Pakistan; ^2^Department of Internal Medicine, Dow University of Health Science, Karachi, Sindh, Pakistan; ^3^Department of Internal Medicine, Ziauddin University, Karachi, Sindh, Pakistan; ^4^Department of Internal Medicine, Dow International Medical College, Karachi, Sindh, Pakistan; ^5^Department of Internal Medicine, GCS Medical College, Hospital and Research Centre, Ahmedabad, Gujarat, India; ^6^Department of Internal Medicine, Karachi Medical and Dental College, Karachi, Sindh, Pakistan; ^7^Department of Internal Medicine, Sri Padmavathi Medical College, SVIMS, Tirupati, Andhra Pradesh, India; ^8^Department of Internal Medicine, Kakatiya Medical College, Warangal, Telangana, India; ^9^Department of Internal Medicine, Sri Ram Murti Smarak Institute of Medical Sciences, Bareilly, Uttar Pradesh, India; ^10^Department of Internal Medicine, Nishtar Medical University, Multan, Punjab, Pakistan *Correspondence: Vikash Kumar Karmani. Email: vikashkarmani@gmail.com

**Keywords:** Blood pressure, intracerebral hemorrhage, ischemic stroke, post-endovascular therapy

## Abstract

**Objective:**

This systematic review and meta-analysis examines the impact of intensive versus standard blood pressure control following post-endovascular therapy in ischemic stroke patients.

**Methods:**

We conducted a systematic review and meta-analysis in accordance with PRISMA (Preferred Reporting Items for Systematic Reviews and Meta-Analyses) guidelines by searching PubMed, Google Scholar, and Cochrane Central databases from inception to December 2023. The outcomes evaluated included symptomatic intracerebral hemorrhage, functional independence (modified Rankin Scale (mRS) score 0–2), death or disability (mRS score 3–6), and health-related quality of life (three-level EuroQoL five-dimensional self-report questionnaire (EQ-5D-3L score). We used the standard mean difference (SMD) with a 95% confidence interval (CI) for continuous outcomes in all studies and used a random-effects model for data synthesis irrespective of heterogeneity. Heterogeneity was assessed using the I^2^ statistics.

**Results:**

We screened 2,000 articles and included four randomized controlled trials (3,635 patients). Intensive blood pressure control affected the health-related quality of life (EQ-5D-3L score) more than standard blood pressure (SMD = -0.22, 95% CI: -0.34 to -0.11, *p* = 0.0002). However, intensive blood pressure control after endovascular therapy did not significantly reduce the risk of intracerebral hemorrhage within 36 hours (risk ratio (RR) = 0.91, 95% CI: 0.70–1.19, *p* = 0.51). Additionally, there was an insignificant improvement in the likelihood of regaining functional independence (mRS score 0–2) at three months (RR = 0.87, 95% CI: 0.73–1.04, *p* = 0.12). Moreover, there was an insignificant increase in the risk of death or disability (mRS score 3–6) at 3 months with intensive blood pressure control compared to standard blood pressure control (RR = 1.18, 95% CI: 0.93–1.51, *p* = 0.18).

**Conclusion:**

In summary, our findings indicate that implementing intensive blood pressure control does not lead to an increased risk of adverse outcomes such as intracranial hemorrhage within 36 hours, compromised functional independence, disability, or mortality 3 months following endovascular therapy. Despite the observed reduction in health-related quality of life reflected in the EQ-5D-3L score, the overall safety profile of intensive blood pressure control compared to standard management suggests its viability as a potential strategy for improving patient outcomes in the context of endovascular therapy.

## Introduction

An ischemic stroke occurs due to cerebral ischemia, which is characterized by reduced or obstructed blood flow to a specific region of the brain, leading to cerebral hypoxia and limited nutrient supply to neuronal cells.^1^ The occlusion is predominantly caused by arterial blockage resulting from thrombotic or embolic events and is often triggered by intravascular thrombus formation or atherosclerotic plaque accumulation in the cerebral vasculature.^2^ Ischemic stroke accounts for more than 85% of all strokes worldwide and remains a leading cause of mortality and morbidity.^3,4^ Its incidence varies globally and has significant regional differences. In recent decades, the global burden of stroke has transitioned from developed to developing countries.^5^ These countries now account for 75% of stroke deaths and 81% of total DALYs (Disability-Adjusted Life Years).^5^

The standard approach to treating acute ischemic stroke caused by intracranial large-vessel occlusion typically involves endovascular thrombectomy or endovascular therapy (EVT), either as a stand-alone procedure or in conjunction with intravenous thrombolysis.^6^ Although positive radiological outcomes are often achieved, a significant number of patients still experience suboptimal functional recovery. Additionally, the associated risks of complications such as symptomatic intracerebral hemorrhage (ICH) and reperfusion injury remain significant.^7,8^ This has led to a growing interest in examining the impact of blood pressure (BP) management on patients undergoing reperfusion therapies for acute ischemic stroke. High BP, an easily modifiable factor, has consistently shown prognostic significance in observational studies associated with endovascular treatment.^9–11^

A previous meta-analysis suggested potential advantages in implementing intensive BP control after EVT.^12^ Furthermore, several observational studies have established a correlation between elevated systolic BP after EVT and adverse clinical outcomes. Overall, this highlights the potential benefits of intensive BP control following EVT procedures.^13–15^

This systematic review and meta-analysis aims to assess the impact of intensive BP management (systolic BP target range: ≥ 100 to ≤ 140 mmHg) compared to standard (or conventional) BP control (systolic BP target range: ≥ 140 to ≤ 185 mmHg) among individuals who experienced an acute ischemic stroke and underwent EVT.

## Methods

**Figure 1.**
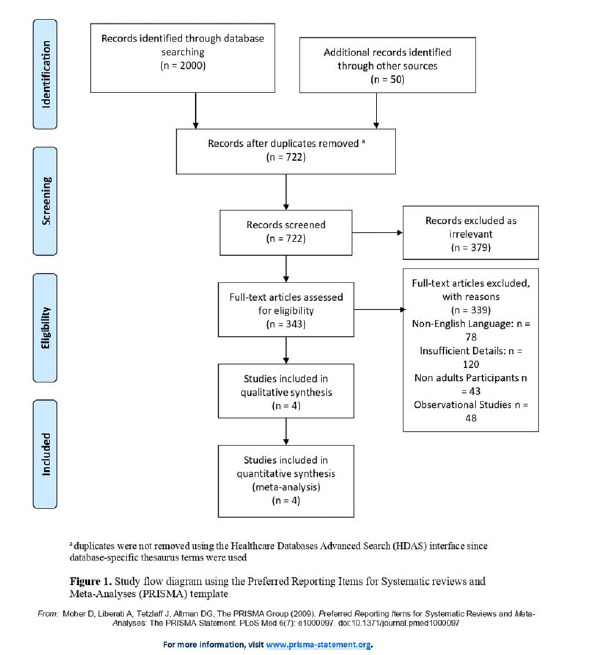
PRISMA flow diagram showing the literature search and study selection process.

This meta-analysis was conducted according to the Cochrane Handbook and PRISMA (Preferred Reporting Items for Systematic Reviews and Meta-Analyses) guidelines (Figure 1).^16,17^ Approval from the Institutional Review Board was not required as only publicly available data were used for the analysis.

### Literature search and study selection

We extensively searched PubMed, Google Scholar, and Cochrane Central databases from inception to December 2023 to collect relevant studies. The search strategy included terms such as “intensive Blood Pressure”, “standard Blood Pressure”, “conventional Blood Pressure”, “ischemic stroke”, and “non-hemorrhagic stroke”. Table S1 shows a detailed search strategy for the included databases. After retrieving the studies, duplicates were identified and removed using the EndNote reference management software, version 20.2.1 (Clarivate Analytics). The reference lists of retrieved trials, previous meta-analyses, and review articles were also examined to include additional relevant studies. Two independent authors (V.K. and B.J.) conducted a thorough review of the remaining articles based on titles and abstracts, followed by a full-text review to select relevant studies. In case of any disagreement, a third reviewer (Z.K.) resolved the dispute.

### Inclusion and exclusion criteria

We included peer-reviewed human studies that met the following inclusion criteria: (1) randomized controlled trials (RCTs); (2) adult patients aged 18 years or older with ischemic stroke who underwent successful EVT and had elevated BP ( ≥ 2 consecutive measurements of systolic BP ≥ 140 mmHg for >10 min) within 3 hours of successful reperfusion, assigned to intensive (systolic BP target < 140 mmHg) or standard (systolic BP target 140–185 mmHg) BP control interventions; (3) studies providing data for at least one of the following outcomes: symptomatic ICH within 36 hours, functional independence (modified Rankin Scale (mRS) score 0–2) at 3 months, death or disability (mRS score 3–6) related to the index stroke within 3 months, and health-related quality of life (HRQoL) on the three-level EuroQoL five-dimensional self-report questionnaire (EQ-5D-3L score) at 3 months.

Exclusion criteria included individuals with heart failure (ejection fraction < 30%), hemorrhagic complications during EVT, severe comorbidities hindering procedural benefits, significant pre-stroke disability (mRS score 3–5), known pregnancy or lactation, anticipated lack of therapy benefits (e.g., advanced dementia or high mortality risk within 24 hours after thrombectomy), and contraindications to alteplase or prescribed antihypertensive drugs. We also excluded any observational studies, narrative reviews, letters, editorials, case series, or conference abstracts.

### Data extraction and quality assessment

Two independent reviewers (V.K. and B.J.) independently extracted data from the included studies, including author name, study year, location and groups, sample size, percentage of male participants, mean age, systolic and diastolic BP, median NIHSS (National Institutes of Health Stroke Scale) score, pre-existing medical conditions, medication use, and relevant outcome variables. A third reviewer (Z.K.) checked the data extraction for accuracy, and any discrepancies were resolved through mutual discussion. The methodological quality of the included RCTs was assessed using the revised Cochrane Collaboration's Risk of Bias tool (RoB-2).^18^ The tool evaluated five domains (randomization and allocation adequacy, blinding, loss to follow-up, selective reporting, and other potential sources of bias) and categorized each into low, high, or unclear risk of bias.^18^

### Statistical analysis

Statistical analysis of the compiled data was conducted using Review Manager, version 5.4.1 (Copenhagen: The Nordic Cochrane Centre, The Cochrane Collaboration, 2014). Random-effects models were used in the final data analysis. Standard mean differences (SMD) for continuous outcomes were pooled using the generic inverse variance. Risk ratios (RR) for dichotomous outcomes were calculated by pooling the extracted data. Heterogeneity between studies was assessed using the Higgins I^2^ statistic test, with heterogeneity greater than 75% considered significant.^19^ To identify the source of heterogeneity, a leave-one sensitivity analysis was performed by systematically excluding each study until heterogeneity either decreased or reached 0%. We did not evaluate publication bias because the number of studies was less than 10.

## Results

### Literature search results

Our literature search initially yielded 2,050 potential articles, which were reduced to 722 after removing duplicates. Title and abstract screening resulted in the exclusion of 379 studies. A thorough full-text assessment of 343 articles for eligibility resulted in the exclusion of 339 articles that did not meet the inclusion criteria. Consequently, four RCTs were included.^20–23^ Figure S1 shows the quality assessment using the revised Cochrane Risk of Bias tool for RCTs.

### Baseline characteristics and quality assessment

All the studies included in this meta-analysis were multicenter RCTs. Among them, one study spanned 15 different countries,^23^, while the others originated from South Korea,^20^ France,^21^ and China^22^, respectively The collective cohort included 3,635 patients who underwent EVT for ischemic stroke, with 1,801 in the intensive BP management group and 1,834 in the standard BP management group. Detailed characteristics of the included studies are outlined in Table 1. The risk of bias for studies assessed using the revised Cochrane tool indicated that all RCTs included in the meta-analysis had a lower risk of bias.

### Clinical outcomes

Figure S2 shows forest plots for the outcomes: (A) symptomatic ICH, (B) functional independence (mRS score 0–2), (C) death or disability (mRS score 3–5), (D) health-related quality of life (EQ-5D-3L score).

#### Symptomatic intracerebral hemorrhage

Our analysis showed that the patients who underwent an intensive BP control regimen had a statistically non-significant decrease in the risk of ICH within 36 hours following EVT for stroke compared with those managed according to the standard BP protocol (RR = 0.91, 95% confidence interval (CI): 0.70–1.19, *p* = 0.51), with no significant heterogeneity detected across studies (I^2^ = 25%, *P* for heterogeneity = 0.26) (Figure S2A).

#### Functional independence (mRS score 0–2)

Our analysis showed that the patients who underwent an intensive BP control regimen had an insignificantly reduced likelihood of regaining functional independence three months after the EVT for stroke compared to the standard BP management group (RR = 0.87, 95% CI: 0.73–1.04, *p* = 0.12), with a significant heterogeneity of 83% found across all studies (I^2^ = 83%, *P* for heterogeneity = 0.0006) (Figure S2B). The sensitivity analysis showed that “Anderson 2018” could be the source of heterogeneity. After excluding this study, heterogeneity between studies was reduced (I^2^ = 40%, *P* for heterogeneity >0.1) (Figure S2B).

#### Death or disability (mRS score 3–6)

Our analysis showed that in patients who underwent intensive BP control, there was an insignificant increase in the risk of death or disability (mRS score 3–6) at 3 months after EVT for stroke compared to the standard BP management group (RR = 1.18, 95% CI: 0.93–1.51, *p* = 0.18), with 79% heterogeneity detected across the studies (I^2^ = 79%, *P* for heterogeneity = 0.009) (Figure S2C). The sensitivity analysis showed that “Anderson 2018” could be the source of heterogeneity. After excluding this study, heterogeneity between studies was reduced (I^2^ = 0%, *P* for heterogeneity >0.1) (Figure S2C).

#### Health-related quality of life (EQ-5D-3L score)

Our analysis showed that the patients who underwent intensive BP control management had significantly reduced health-related quality of life, as indicated by a lower EQ-5D-3L score at 3 months after EVT for stroke compared to the standard BP management group (SMD = -0.22, 95% CI: -0.34 to -0.11, *p* = 0.0002), with no significant heterogeneity detected across the studies (I^2^ = 0%, *P* for heterogeneity = 0.67) (Figure S2D).

## Discussion

Management of BP following EVT for acute ischemic stroke is intricate. Some stroke centers consider reperfusion status when setting BP thresholds, although conclusive evidence justifying this approach is lacking.^24,25^ Lowering BP may mitigate ischemia–reperfusion lesions, including intraparenchymal hemorrhage, but could potentially increase the risk of extensive infarct growth in cases of incomplete or unsuccessful reperfusion.^26^ Current international guidelines lack specificity regarding targets based on reperfusion status. They recommend a systolic BP target of less than 185 mmHg before EVT and 180 mmHg within the first 24 hours after the procedure.^27,28^ However, new evidence suggests potential risks associated with systolic BP levels as low as 140 mmHg after successful reperfusion.^24^ Notably, clinical practice in the USA, as revealed by the StrokeNet survey, often adopts a systolic BP target in the range of 120–139 mmHg for reperfused patients, while allowing permissive hypertension in non-reperfused patients.^25^ This consensus is guided by current BP management practices, but remains to be confirmed by meta-analysis. Therefore, this study is the first aimed at assessing systolic BP targets in the post-reperfusion phase of endovascular treatment of acute ischemic stroke.

According to our meta-analysis, the risk of symptomatic ICH is comparable between the intensive and conventional BP management groups. This is inconsistent with the findings of previous observational studies,^13,29^ registry studies,^30^ and systematic reviews^11,27^ that established an association between elevated systolic BP and adverse functional outcomes or the occurrence of intraparenchymal hemorrhage. The previous studies suggested that lower systolic BP targets ranging from < 130 mmHg to < 160 mmHg correlated with better outcomes.^30,31^ However, our results are consistent with the outcomes of previous RCTs such as BP-OPTIMAL,^20^ BP-TARGET trials,^21^ and ENCHANTED2/MT,^22^ but differ from the ENCHANTED trial,^23^ which indicated a significant reduction in the risk of ICH with intensive BP control. In particular, the variations in systolic BP targets across these trials (e.g., < 120 mmHg,^22^ < 130 mmHg,^21^ < 140 mmHg,^20^ and 130–140 mmHg^23^) should be considered when interpreting the results.

This meta-analysis indicated that the risk of ICH may be marginal and not significantly different as long as BP is below 180 mmHg and current guidelines are followed.^32,33^. High BP in acute stroke patients could increase the risk of ICH, possibly in response to acute stress. Activation of the sympathetic nervous system and the renin–angiotensin axis has been linked to this increase in BP.^34^ Brain swelling may contribute and trigger a compensatory increase in BP to maintain cerebral perfusion pressure.^35^ A previous trial indicated that the higher frequency of malignant cerebral edema in the intensive management group suggests potential harm to the cerebral microcirculation with more aggressive BP control.^23^ Interestingly, in the conventional management group without active BP lowering, lower mean 24-hour BP correlated with better outcomes.^21^ This discrepancy with observational studies highlights the challenge of controlling systolic BP, possibly influenced by variability in the first 24 hours after EVT, which correlates with symptomatic intraparenchymal hemorrhage rather than absolute BP levels.^36^ While high BP may increase the risk of ICH, the adverse outcomes in patients with high BP in observational studies could be attributed to an appropriate physiological response to severe stroke, casting doubt on the safety of targeting such low BP levels.^13,29^

Our study also revealed that intensive BP management following EVT for ischemic stroke is associated with worse functional outcomes, as represented by a decreased likelihood of functional independence and an increased likelihood of disability or death at 3 months, compared to conventional BP control. Although this difference lacks statistical significance, it is consistent with the findings of previous RCTs^20–23^ and contrasts with results from observational studies.^13,29^ The optimal systolic BP target after reperfusion remains debatable, with reported differential effects, including a lower odds of symptomatic ICH for systolic BP < 120 mmHg but a higher odds for mortality.^37^ This evidence supports the increased likelihood of mortality associated with low baseline systolic BP, before any reperfusion therapy, compared to systolic BP levels ranging from 140 mmHg to 160 mmHg.^38^

The observed effects may be attributed to damaged or oligemic areas in the ischemic brain that lack sufficient autoregulatory function to compensate for a sudden drop in BP. Intensive BP lowering may exacerbate ischemic injury by further reducing blood flow to such areas. Additionally, successful reperfusion after EVT may not guarantee resolution of venous post-capillary thrombosis, also known as no-reflow, leading to unfavorable outcomes.^39^ Therefore, the potential benefit of reducing ICH through intensive BP reduction could be counteracted by hypoperfusion of the ischemic penumbra, emphasizing the need for careful consideration of BP management strategies to balance potential risks and benefits.

After experiencing a stroke, individuals often face persistent challenges that affect physical, mental, and social functioning.^40^ A crucial aspect of post-stroke care is the assessment of HRQoL, defined as an individual's perceived physical and mental health over time, emphasizing the impact of health status on overall quality of life.^41^ Existing observational studies suggest that maintaining systolic BP within the target range of 140–150 mmHg is associated with favorable long-term outcomes.^23^ Surprisingly, our study reveals a negative correlation between intensive BP management and HRQoL, as evidenced by lower EQ-5D-3L scores. One possible reason for the inverse association found between systolic BP and prognosis in observational studies could lie in greater increases in BP in individuals with a large acute ischemic stroke volume, severe ischemia, reduced likelihood of reperfusion, leading to BP-lowering treatment, and ultimately resulting in poorer long-term outcomes.^42,43^

In contrast, significant acute ischemic stroke volume may lead to focal hypoperfusion clusters that persist after successful EVT, potentially progressing to infarction following intensive systolic BP lowering and therefore accounting for a significant association between intensive BP management and lower HRQoL in our study. Understanding the impact on HRQoL is pivotal for comprehensive stroke management as patients seek not only survival and functional recovery but also a good quality of life. As rehabilitation goals are consistent with achieving a good HRQoL, our study highlights the evolving patient-centered nature of the healthcare approach. Identifying and addressing HRQoL reduction can inform tailored interventions and support systems, ultimately enhancing the overall recovery from stroke.

## Strengths And Limitations

The existing international guidelines on BP management lack specific targets based on reperfusion status after EVT for acute ischemic stroke. Previous observational studies and RCTs have yielded conflicting findings regarding the impact of intensive BP management in EVT-treated ischemic stroke patients. This meta-analysis, particularly the first of its kind, focuses on evaluating post-reperfusion systolic BP targets in the endovascular era of acute ischemic stroke treatment. Key strengths include a large sample size, international representation, and robust methodologies, enhancing the generalizability of the results and potential influence on global clinical practice. The exclusive inclusion of RCTs further adds credibility and resolves the debate surrounding this issue.

However, our study has some limitations that deserve consideration. Firstly, the meta-analysis included a relatively small number of RCTs with a brief follow-up period, which may have contributed to statistical insignificance in some outcomes. Secondly, the significant heterogeneity of the results was due to varying systolic BP targets ( < 120 to 140 mmHg) for intensive management, variations in time spent within the systolic BP target range, and confounding factors such as stroke volume, severity (ASPECT (Alberta Stroke Programme Early Computed Tomography) and NIHSS scores), baseline BP, and medication use. The use of different drugs such as nicardipine (calcium channel blocker)^21^ and urapidil (α-blocker)^22^ may have contributed to this heterogeneity. Thirdly, the study did not address the role of antihypertensive drugs that affect cerebral blood flow, potentially influencing outcomes (calcium channel blockers, which are believed to have neuroprotective properties, or β-receptor antagonists, which could be harmful by decreasing cardiac output).^44^ Moreover, the study did not determine the role of intensive BP lowering in patients with low ASPECT scores or severe hypertension, as the patients with low ASPECT scores or baseline systolic BP greater than 185 mmHg rarely underwent EVT in the included trials.^21,23^ Therefore, careful interpretation of the results is essential.

## Future Directions

These results pave the way for future research to examine whether maintaining a stable BP, rather than adhering to a specific systolic BP target, could prevent adverse outcomes after successful EVT. Further research is needed to assess the various antihypertensive drugs used in this context. Trials specifically focusing on patients with large ischemic cores could shed light on the benefits of intensive BP reduction. While optimal thresholds or systolic BP ranges remain undefined, ongoing and upcoming trials such as BEST-II (NCT04116112), DETECT (NCT04484350), CRISIS I (NCT04775147), and HOPE (NCT04892511), as well as the reactivation of ENCHANTED2/MT with a revised protocol, have the potential to address these issues, reinforce our findings, and establish an optimal level of BP control for patients who achieve successful reperfusion after EVT.

## Conclusion

In summary, among patients with acute ischemic stroke and elevated BP ( ≥ 140 mmHg) after successful reperfusion by EVT, more aggressive BP reduction to a target systolic level below 140 mmHg showed no significant impact on the risk of ICH within 36 hours, functional independence (mRS score 0–2), disability (mRS score 3–5), or death at 3 months after EVT compared to standard BP management (140–185 mmHg). Additionally, intensive BP control resulted in a significant worsening of health-related quality of life (EQ-5D-3L score) compared to standard management. These findings suggest that intensive BP management should be avoided after successful EVT for acute ischemic stroke. Furthermore, the optimal systolic BP target after endovascular thrombectomy for acute ischemic stroke patients remains to be defined.

### Authors' contribution

**B.J.**, **V.K.**, **Z.K.**, **A.R.**, **Y.M.**, **M.M.**, and **M.C.**: Responsible for the concept and design of the study. **H.K.**, **M.M.**, **M.C.**, **A.S.**, and **S.Z.**: Contributed to data acquisition. **Z.K.**, **Y.M.**, **V.K.**, **A.S.**, **H.K.**, and **S.Z.**: Conducted the statistical analysis. **V.K.**, **B.J.**, **I.J.**, **Z.K.**, and **Y.M.**: Performed the DNA extraction and interpreted the results. **Y.M.**, **V.K.**, **Z.K.**, **I.J.**, **B.J.**, and **A.R.**: Analyzed the data and drafted the manuscript. All authors critically revised the manuscript, approved the final version to be published, and agreed to be accountable for all aspects of the work.

### Competing interests

The authors have no conflicts of interest to declare.

### Data availability statement

All data generated or analyzed during this study are included in the published article (and its supplementary information files).

## Figures and Tables

**Table 1.** Baseline characteristics of the included trials.

**Author, year**	**Nam et al., 2023^21^**		**Mazighi et al., 2021^22^**		**Yang et al., 2022^23^**		**Anderson et al., 2019^24^**	

Study groups	Intensive BP control	Standard BP control	Intensive BP control	Standard BP control	Intensive BP control	Standard BP control	Intensive BP control	Standard BP control

Study location	South Korea		France		China		International	

Study design	Multicenter RCT		Multicenter RCT		Multicenter RCT		Multicenter RCT	

Sample size	155	150	158	160	407	409	1,081	1,115

Male, *n* (%)	92 (59.4)	88 (59.9)	81 (51.0)	72 (45.0)	249 (61.0)	257 (63.0)	680 (62.9)	681 (61.1)

Age (years), mean ± SD	73.2 ± 12.1	72.9 ± 10.8	77 ± 4.7	76 ± 5.0	68 ± 12.0	67 ± 12.0	66.7 ± 12.4	67.1 ± 12.0

Systolic BP (mmHg), mean ± SD	–	–	155.0 ± 26.0	152.0 ± 25.0	158.1 ± 25.0	158.7 ± 23.0	165.4 ± 9.1	165.2 ± 9.2

Diastolic BP (mmHg), mean ± SD	–	–	86.0 ± 18.0	85.0 ± 15.0	89.4 ± 16.0	89.5 ± 15.0	91.2 ± 11.6	90.7 ± 11.3

NIHSS score, median (IQR)	–	–	18 (12–20)	17 (13–20)	15 (10–20)	15 (10–20)	7 (4–12)	8 (4–12)

HTN, *n* (%)	121 (78.1)	110 (74.8)	110 (70.0)	113 (70.6)	276 (66.0)	261 (64.0)	773 (71.7)	795 (71.4)

DM, *n* (%)	65 (41.9)	62 (42.2)	34 (21.9)	33 (20.7)	81 (20.0)	82 (20.0)	230 (21.3)	266 (23.9)

Smoking, *n* (%)	39 (25.2)	29 (19.7)	19 (13.8)	24 (16.4)	–	–	218 (20.2)	226 (20.3)

Hypercholesterolemia, *n* (%)	–	–	59 (38.5)	55 (34.8)	14 (3.0)	13 (3.0)	120 (11.1)	129 (11.6)

Previous stroke, *n* (%)	36 (23.2)	30 (20.4)	25 (16.0)	21 (13.2)	107 (26.0)	139 (34.0)	205 (19.0)	209 (18.7)

Atrial fibrillation, *n* (%)	77 (49.7)	69 (46.9)	–	–	84 (21.0)	98 (24.0)	140 (13.0)	172 (15.5)

CAD, *n* (%)	18 (11.6)	16 (10.9)	–	–	51 (13.0)	59 (14.0)	154 (14.3)	155 (13.9)

VHD, *n* (%)	–	–	–	–	16 (4.0)	17 (4.0)	42 (3.9)	52 (4.7)

Medication use								

Statins and other lipid-lowering drugs, *n* (%)	–	–	–	–	30 (7.0)	30 (7.0)	154 (14.3)	184 (16.5)

Antiplatelet/aspirin, *n* (%)	–	–	44 (28.2)	37 (23.1)	34 (8.0)	39 (10.0)	174 (16.1)	212 (19.0)

Anticoagulants, *n* (%)	–	–	34 (21.7)	34 (21.2)	20 (5.0)	20 (5.0)	14 (1.3)	15 (1.3)


BP: blood pressure, RCT: randomized controlled trial, *n*: number, SD: standard deviation, NIHSS: National Institutes of Health Stroke Scale, IQR: interquartile range, HTN: hypertension, DM: diabetes mellitus, CAD: coronary artery disease, VHD: valvular heart disease.
